# Collagen peptide supplementation before bedtime reduces sleep fragmentation and improves cognitive function in physically active males with sleep complaints

**DOI:** 10.1007/s00394-023-03267-w

**Published:** 2023-10-24

**Authors:** Craig Thomas, Ruth N. Kingshott, Kirsty M. Allott, Jonathan C. Y. Tang, Rachel Dunn, William D. Fraser, Josh Thorley, Nicolina Virgilio, Janne Prawitt, Eef Hogervorst, Jakob Škarabot, Tom Clifford

**Affiliations:** 1 School of Sport, Exercise and Health Sciences, Loughborough University, Loughborough, Leicestershire, LE11 3TU UK; 2 Sheffield Children’s NHS Foundation Trust, The Sleep House, Sheffield, UK; 3 Bioanalytical Facility, Norwich Medical School, University of East Anglia, Norfolk and Norwich University Hospital Norfolk, Norwich, UK; 4 Rousselot BV, Ghent, Belgium; 5 Clinical Biochemistry, Departments of Laboratory Medicine and Departments of Diabetes and Endocrinology Norfolk, Norwich University Hospital NHS Foundation Trust, Norwich, UK

**Keywords:** Recovery, Insomnia, Dietary supplement, Protein, Exercise

## Abstract

**Purpose:**

The primary aim of this study was to examine whether a glycine-rich collagen peptides (CP) supplement could enhance sleep quality in physically active men with self-reported sleep complaints.

**Methods:**

In a randomized, crossover design, 13 athletic males (age: 24 ± 4 years; training volume; 7 ± 3 h·wk^1^) with sleep complaints (Athens Insomnia Scale, 9 ± 2) consumed CP (15 g·day^1^) or a placebo control (CON) 1 h before bedtime for 7 nights. Sleep quality was measured with subjective sleep diaries and actigraphy for 7 nights; polysomnographic sleep and core temperature were recorded on night 7. Cognition, inflammation, and endocrine function were measured on night 7 and the following morning. Subjective sleepiness and fatigue were measured on all 7 nights. The intervention trials were separated by ≥ 7 days and preceded by a 7-night familiarisation trial.

**Results:**

Polysomnography showed less awakenings with CP than CON (21.3 ± 9.7 vs. 29.3 ± 13.8 counts, respectively; P = 0.028). The 7-day average for subjective awakenings were less with CP vs. CON (1.3 ± 1.5 vs. 1.9 ± 0.6 counts, respectively; P = 0.023). The proportion of correct responses on the baseline Stroop cognitive test were higher with CP than CON (1.00 ± 0.00 vs. 0.97 ± 0.05 AU, respectively; P = 0.009) the morning after night 7. There were no trial differences in core temperature, endocrine function, inflammation, subjective sleepiness, fatigue and sleep quality, or other measures of cognitive function or sleep (P > 0.05).

**Conclusion:**

CP supplementation did not influence sleep quantity, latency, or efficiency, but reduced awakenings and improved cognitive function in physically active males with sleep complaints.

## Introduction

Insomnia, defined by nocturnal sleep disturbance (e.g., difficulty falling asleep and staying asleep) and functional daytime impairment, is one of the most common sleep disorders [1]. Indeed, survey data has revealed that 5–20% of adults suffer from insomnia [2, 3]. Insomniacs have reduced quality of life, impaired cognition and mood, and poorer physical and mental health [4]. The first line of treatment for insomnia is cognitive behavioural therapy [5, 6], which is often combined with education on lifestyle (e.g., diet, exercise, substance use) and environmental (e.g., light, noise, temperature) strategies that support or interfere with sleep [5, 6].

As several nutrients interact with neurotransmitters that help regulate the sleep–wake cycle, there is a growing interest in dietary strategies that promote sleep [7]. For example, recent research has shown that supplementation with phytochemicals such as tart cherries [8], or kiwi fruit [9], which are both rich in the sleep promoting hormone melatonin, may enhance sleep quality in humans. In addition, high glycaemic index carbohydrates [10, 11] and α-lactalbumin [12, 13] before bedtime may reduce sleep onset latency, increase sleep efficiency, and extend total sleep time, via their effects on the amino acid tryptophan. Increasing the ratio of tryptophan to other large amino acids in plasma may stimulate the release of brain serotonin [14–16], which is converted to melatonin. This subsequently decreases nocturnal core body temperature and promotes sleep [17].

The non-essential amino acid, glycine, is another nutrient that may improve sleep by activating *N*-methyl-D-aspartate (NMDA) receptors in the suprachiasmatic nucleus (SCN). In animals, activating the NMDA receptors in the SCN increased blood flow and decreased nocturnal core body temperature, increasing the propensity for sleep [18]. In humans, 3 g of glycine taken 1 h before bedtime reduced daytime sleepiness, shortened polysomnographic sleep onset latency (SOL), and improved subjective sleep quality and memory task performance in individuals with sleep complaints [19]. Furthermore, L-serine, a precursor for glycine, taken 30 min before bedtime improved subjective sleep quality, and led to a modest decrease in actigraphic awakenings compared to a placebo [20]. Hence, glycine ingestion is now recommended as a sleep aid [21–23].

Collagen peptides (CP) contain high amounts of glycine, as well as proline and hydroxyproline [24], and are increasingly examined for their potential therapeutic health effects in athletic and clinical populations. While there are currently no studies examining the effects of CP on sleep, their high glycine content suggests they could have a positive impact, especially for those with sleep complaints. CP may also be more effective than glycine alone due to its potential effects on nerve growth factors associated with sleep regulation. Recent animal studies show that peptides from orally ingested collagen hydrolysates reach the cerebrospinal fluid and increase the hippocampal expression of nerve growth factors, including brain derived neurotrophic factor (BDNF) [25, 26], which, when administered to animals, increased non-rapid eye movement sleep [27]. That human and animal studies show BDNF is diminished in poor sleepers [28, 29] and after sleep restriction [30, 31], suggests interventions promoting BDNF expression may impact sleep. Taken together, these findings suggest that CP supplementation has the potential to positively influence sleep quality.

Recent research suggests that athletic populations (e.g., student athletes, national, and elite level athletes) may sleep worse than the general population [32–34]. Indeed, studies have shown that student athletes have more variable sleep patterns [35], tend to sleep less [32] and report high levels of daytime sleepiness [36]. There are several possible reasons why athletic populations may experience increased daytime sleepiness and worse sleep quality; heightened or excessive physical activity, high supplement intake (e.g., caffeine), increased post-exercise body temperature and/or elevated levels of fatigue and pain, and increased sympathetic nervous system activation, could all feasibly negatively impact sleep quality [37–39]. Consequently, there is a growing interest in dietary strategies that could enhance sleep quality in this population. Supplementation with CP is increasingly popular amongst athletic populations, as studies show daily supplementation may improve tendon health and reduce injury risk [40, 41], accelerate post-exercise recovery [42] and promote muscle hypertrophy [43]. Thus, the primary aim of this study was to examine whether CP supplementation could enhance sleep quality in physically active men with self-reported sleep complaints. We hypothesized that CP would lower nocturnal core body temperature, enhance sleep quality — as measured by polysomnographic changes, and that this would enhance cognitive performance and modify markers of endocrine and immune disruption.

## Methods

### Sample size estimation

Our sample size was determined from an a priori power analysis (G*power 3.1.9.2, Microsoft Windows [44]) for our two primary outcome measures, SOL, and sleep efficiency (SE), as measured by polysomnography (PSG). Based on previous research [10, 19, 45], we used a mean difference of 10 min (10 min SD) for SOL, and 4% for SE (4% SD) to estimate our sample size. With two tails, and an alpha of 0.05, it was calculated that we needed ≥ 10 participants to achieve 80% statistical power.

### Participants

Thirteen athletic males with sleep complaints provided written informed consent to participate in this study; characteristics are presented in Table 1. Height and body mass were recorded at familiarisation. Inclusion criteria; 1) males 18–35 years; 2) Athens Insomnia Scale (AIS) score ≥ 6, indicating difficulty sleeping; 2) morning-eveningness questionnaire (MEQ) score of 31–69, indicating neither extreme morning or evening person; 3) scheduled exercise ≥ 3 occasions per week in the past 6 months. Participants completed a health history questionnaire and were excluded if they used sleep medications, had a diagnosed sleep disorder, reported excessive use of substances that negatively affect sleep such as drugs, alcohol, or smoking, regularly used anti-inflammatory medications (within 2 weeks of participation), had a food allergy, or any other contraindication to the study procedures. The protocol received ethical approval from Loughborough University School of Sport, Exercise, and Health Sciences Ethics Committee (ethics number: 1781) and was conducted in accordance with the Declaration of Helsinki. The study was pre-registered on the Open Science Framework: osf.io/vjf8y.

**Table 1:** Physical characteristics and activity levels of study participants

Variable	*Mean* ± *SD*
Age (years)	24 ± 4
Height (m)	1.8 ± 0.1
Body mass (kg)	79.2 ± 12.6
AIS	9 ± 2
MEQ	50 ± 8
Exercise training frequency (n/week)	5 ± 2
Exercise training volume (h/week)	7 ± 3

*AIS* Athens Insomnia Scale; *MEQ* Morning-eveningness questionnaire

### Experimental design

Prior to the supplementation trials, participants completed a familiarisation trial, in which they had their physical activity and sleep monitored for 7 days and nights in their home. This was to characterise exercise and sleep schedules (i.e., bedtimes and wake times) for the supplementation trials, and to familiarise them with the protocol and equipment. No supplements were consumed for these 7 days. Sleep was monitored via actigraphy and subjective sleep quality for 7 days, and then PSG on the final 2 nights (nights 6 and 7).

Following this familiarisation trial, participants were randomised, in a double-blind manner, to consume a 200 ml bolus of a CP based or a placebo control (CON) drink 1 h before bedtime for 7 days (trials were separated by ≥ 7 days). The trial order was randomly generated using online software (Graphpad Prism, CA, US). We provided CP for 7 days as this duration of intake has previously been shown to positively influence physiological markers [42, 46].

Sleep monitoring during both trials was the same as the familiarisation trial. Surveys to assess fatigue and sleepiness were also completed daily. On day 7, participants reported to the lab in the evening (≥ 16:00), had a venous blood and urine sample collected, consumed a telemetric temperature pill (E-celsius Performance Pill, Body Cap, France) and performed a battery of cognitive function tests. Although the timing of pill ingestion (1 – 12 h before) does not appear to interfere with the validity of the sensor [47], consistent with previous studies, we provided the pill 5–8 h before our first recording [46, 48], to ensure passage from the stomach. These telemetric pills have been shown to have excellent reliability and validity [49] and are considered the industry standard for temperature monitoring [50]. Participants returned to the lab after an overnight fast on the morning of day 8 (08:00–10:00) to have another blood and urine sample collected and to repeat the cognitive function tests. Participants were instructed to keep their bedtime and wake times the same as in their familiarisation week. On day 7 of both trials, participants followed a low protein diet and consumed a standardised evening meal. No exercise was permitted on day 7; participants were instructed to replicate their dietary intake and physical activity patterns recorded during the familiarisation week.

### Supplementation

Participants consumed 15 g/day of CP derived from bovine hide or an inert, taste matched, CON as a 200 ml liquid bolus, without food, ~ 1 h before their scheduled bedtime (as recorded in the familiarisation trial) for 7 days. A 15 g CP dose was selected as it contains ~ 3.5 g of glycine, an amount shown to enhance sleep quality [19, 51]. The dose was consumed 1 h pre-bed, as (1) a previous study with glycine showed this improved polysomnographic sleep [19] and, (2) plasma glycine concentrations peak 30–60 min post CP intake [52]. Both supplements were provided in identical opaque bottles by Rousselot BV (Ghent, Belgium). The investigators remained blinded until data analysis was completed. No participants reported adverse effects from the supplements.

### Athens insomnia scale and morning-eveningness questionnaire

The AIS is an eight-item survey that assesses sleep difficulty in the past month; a score of ≥ 6 is indicative of poor sleep [53]. The AIS is based on diagnostic criteria from the International Classification of Diseases Tenth Revision (ICD-10) and has excellent reliability and validity in adult populations [53, 54]. It also has excellent reliability in athletic populations [55]. The MEQ measures chronotype, differentiating individuals into morning and evening types [56]. A score of < 31 indicates an extreme evening type; a score of > 69 indicates an extreme morning type. We excluded either extreme morning or evening types to increase the homogeneity of our sample.

### Sleep and training diaries, and actigraphy

Participants used sleep diaries to self-report their time in bed, bedtime, SOL, number of awakenings, length of awakenings, wake up time, and get up time during the trials. Any day-time naps were also recorded. Training diaries were used to record the intensity and volume of scheduled exercise completed each day. Intensity (1 = very low intensity, 10 = maximum intensity) was multiplied by duration (min) to give a composite score of training load [57]. Participants also wore wristwatch actigraphs (ActiGraph GT9X Link, Actigraph, FL, US) on their non-dominant wrist to measure physical activity levels and sleep. The participants were instructed not to remove the watch apart from to shower or bathe; if the watch was removed at any other point, they were instructed to record this in their sleep diaries. Actigraphy data was sampled at 60 s epochs and analysed using the Cole Kripke algorithm [58] on ActiLife software (ActiGraph, FL, US). Bedtime and wake up times from the sleep diaries were used for data analysis. Sleep variables analysed from actigraphy were time in bed (TIB), total sleep time (TST), SOL, SE, wake after sleep onset (WASO) and average wake length. Physical activity was analysed as total time (min) spent in moderate to vigorous activity using the cut off points suggested by Freedson and colleagues [59].

### Polysomnography

Polysomnographic sleep was measured on nights 6 and 7 of each trial with an ambulatory monitor (Embletta MPR with ST + Proxy, Natus Medical, CA, US). Night 6 served as a familiarisation collection only and was not used for analysis. Six electroencephalogram (EEG) channels (F3-M2; F4-M1; C3-M2; C4-M1; O1-M2 and O2-M1), two electrooculogram (EOG) channels (LEOG-M2 and REOG-M1), a submental chin electromyogram (EMG) (3 EMG electrodes) and the PSG unit were fitted in the lab ~ 6–8 h before sleep. All EEG sites were located using the international 10–20 system. The PSG was scheduled to start recording 30 min prior to bedtime. All recordings were collected at home; participants were instructed to sleep in the same bed, in the same environmental conditions and expose themselves to the same mental stimulation (e.g., phone use, television) in the 1 h before bedtime on each trial. Any deviances were reported in their subjective sleep diaries. Home-based PSG is considered as valid and reliable as clinic-based PSG set ups [60, 61]. Recordings were subsequently exported in EDF format and downloaded into Domino software v3.0.0.3 (SOMNOmedics, Germany) where they were manually scored in 30 s epochs using the standard adult criteria from the American Academy of Sleep Medicine (AASM) [62] by registered sleep physiologists with ≥ 20 years’ experience. Each recording was labelled with a different non-identifiable code so that the scorers (RNK and KMA) were blinded to participant identity and trial. As with actigraphy, lights out was marked as the time participants first attempted to go to sleep and lights on was marked as the time participants woke up for the final time (both from sleep diaries). To assess intra-rater reliability, 3 blinded PSG recordings were scored twice by RNK. To assess interrater reliability, KMA independently scored 3 PSGs previously scored by RNK. Each recording was scored for all outcomes presented in Table 2.

**Table 2:** Polysomnography data for the control and collagen peptides trials

Variable	Control	CP	P value	Effect size	95% CI
Time in bed (min)	493.4 ± 41.3	492.6 ± 48.3	0.912	0.032	− 15.1 to 16.7
Total sleep time (min)	449.2 ± 46.1	454.9 ± 49.4	0.388	0.250	− 19.6 to 8.26
Sleep latency (min)	14.2 ± 10.7	12.8 ± 9.2	0.578	0.160	− 3.85 to 6.57
Sleep efficiency (%)	91.0 ± 4.2	91.9 ± 3.7	0.376	0.257	− 2.98 to 1.22
Sleep latency N2 (min)	18.5 ± 11.6	14.9 ± 8.9	0.245	0.342	− 2.79 to 9.84
Deep sleep latency (min)	29.6 ± 14.1	26.7 ± 9.4	0.418	0.235	− 4.54 to 10.1
REM sleep latency^ (min)	79.9 ± 26.8	76.8 ± 26.4	0.450	0.258	− 10.0 to 25.2
Total sleep period^ (min)	479.1 ± 44.7	479.7 ± 55.7	1.000	0.000	− 16.5 to 15.0
Sleep stage change (count)	113.8 ± 42.1	100.7 ± 30.8	0.188	0.391	− 7.50 to 33.8
Sleep stage change^ (count/hr)	13.8 ± 4.9	12.8 ± 3.3	0.477	0.242	− 1.50 to 3.35
Wakening’s^ (count)	29.3 ± 13.8	21.3 ± 9.7	0.028*	0.782	1.00 to 16.0
Wakening’s^ (count/hr)	4.0 ± 1.9	2.9 ± 1.2	0.028*	0.782	0.15 to 2.00
Wake after sleep onset^ (min)	30.0 ± 21.1	24.4 ± 16.9	0.272	0.359	− 4.46 to 16.0
REM stage sleep# (%)	22.4 ± 3.2	21.6 ± 5.7	0.624	0.140	− 2.52 to 4.02
N1 stage sleep# (%)	5.5 ± 2.5	4.6 ± 1.8	0.109	0.486	− 0.22 to 1.90
N2 stage sleep# (%)	44.2 ± 5.9	45.6 ± 4.3	0.262	0.329	− 4.00 to 1.20
N3 stage sleep# (%)	28.0 ± 5.7	28.2 ± 6.4	0.833	0.042	− 3.13 to 2.73
Light sleep# (%)	49.7 ± 6 .9	50.2 ± 5.2	0.691	0.114	− 3.57 to 2.45
Arousal^ (count)	62.4 ± 41.8	53.4 ± 23.0	0.266	0.379	− 4.50 to 21.5
Arousal^ (count/hr)	8.5 ± 6.0	7.4 ± 3.0	0.533	0.212	−0.95 to 2.40

*REM* rapid eye movement sleep, *CP* collagen peptides

#Data indicates % of total time spent in specific sleep stage. 95% CI = 95% confidence interval for the difference in means

*Indicates statistically significant difference between trials.

^Indicates effects size is rank biserial correlation (all others are Hedges g), and 95% CI is Hodges-Lehman for median of the difference

### Subjective surveys

Subjective sleep quality was recorded with a 7-point Likert scale, where 1 = extremely poor and 7 = extremely good [23]. Sleepiness was measured with the Karolinska Sleepiness Scale (KSS), where 1 = extremely alert, and 10 = extremely sleepy, can’t stay awake [63]. Fatigue was measured with a 100 mm visual analogue scale, where 0 mm = not fatigued at all and 100 mm = extremely fatigued [64]. Sleep quality was recorded upon waking, while KSS and fatigue scores were recorded upon waking, at 14:00 and 1 h before bedtime. These were recorded daily with 7-day averages used for analysis.

### Cognitive function

Participants completed a series of cognitive tests that took approximately 10 min on a laptop. All tests were preceded by 3–5 practice trials. The first test was simple reaction time (RT), where participants were instructed to press the spacebar when a green circle appeared on a blank screen. The second test was choice RT, where participants were presented with right or left arrows and were required to press the arrow key that matched the direction on the screen. Average and fastest reactions times of correct responses (ms) were analysed for both; there were 10 and 14 attempts for simple and choice RT, respectively. Participants then completed the Digit Span Test, which measures attention and short-term recall [65]. To complete this test, a sequence of 3–14 digits were flashed on the screen and then participants were instructed to type the digits in the correct order. The length of the longest correct span over 12 trials was used for analysis. Finally, participants completed the Stroop Color Word Test, which measures attention and processing speed, but primarily the ability to inhibit cognitive interference [66, 67]. For the baseline level, participants were presented with names of colours (written in black) on a white background and instructed to correctly identify the name of the colour by pressing the right or left arrow. For the interference level, participants were presented with colored names on a white background and were required to correctly identify the font color, ignoring the incongruent colour named on the screen (e.g., if “green” is written in red font, then red is the correct answer). Participants had to respond as quickly as possible using left or right arrow keys. Outcomes were the proportion of correct responses, and average RT of correct responses (ms), for the baseline (14 trials) and interference (20 trials) levels.

### Dietary control

Participants recorded their dietary intake on day 7 of the familiarisation trial and were instructed to replicate this intake on the two supplementation trials. On this day they were instructed to limit their intake of protein-rich foods (a list was provided), avoid consuming any caffeine after 11:00, and attend the lab ≥ 2 h post-prandial. Participants were given a low-protein evening meal to consume at home consisting of 1 × 250 g pack of White Golden Vegetable Rice (Tesco, PLC, Herts, UK) and 2 × 37 g Nutrigrain Blueberry bars (Kelloggs, Michigan, US). After this meal they avoided any food or fluids other than water and their respective supplements 1 h before bedtime until after their lab visit on day 8.

### Blood and urine samples

Blood and urine samples were collected the evening of day 7 and the morning of day 8. Urine samples were collected in a plastic container; venous blood samples were obtained via venipuncture into 1 × 10 ml vacutainer for serum and 1 × 10 ml vacutainer coated with EDTA to obtain plasma. The EDTA vacutainer was immediately centrifuged at 3000 ×*g* (4 °C) for 10 min, while the serum vacutainer was left to stand for 30 min prior to centrifugation. Urine samples and plasma and serum supernatants were subsequently stored in aliquots at − 80º.

Samples were analysed for high sensitivity c-reactive protein (hs-CRP), interleukin-6 (IL-6), cortisol, and urine samples for normetanephrine, as these are sensitive to changes in sleep quality [68]. IL-6 was analysed in plasma using a commercially available ELISA kit (R and D Systems, MN, US); the inter-assay coefficient of variation (CV) was < 6.9%.

### Measurements of serum cortisol and urine normetanephrine by LC–MS/MS

Serum cortisol and urine normetanephrine were measured using Liquid chromatography tandem mass spectrometry (LC–MS/MS) methods by a Waters Acquity I-class UPLC system coupled to the Xevo TQ-XS tandem mass spectrometer (Waters Corp., Milford, MA, USA) operated in positive electrospray mode. Serum cortisol was extracted using the Extrahera™ automation system (Biotage, Uppsala, Sweden) under positive pressure supplied by a nitrogen generator. In a 96-position 2 mL deep well plate, 200 μL of calibration standards (Chromsystems, München, Germany NIST SRM971 traceable), quality control materials, and serum samples were added to each well with 50 μL internal standard solution containing cortisol-d4 (IsoSciences, King of Prussia, PA, USA). 200 μL of isopropanol:water 50:50 (v/v) was then added to dissociate the binding proteins. After mixing, the samples were loaded onto ISOLUTE® supported liquid extraction (SLE +) 400 μL plate (Biotage). Elution was carried out by adding two cycles of 750 µL of methyl tertiary butyl ether (MTBE). The eluents were collected into a corresponding deep well plate. Positive pressure was applied at each stage to remove residual solvent. Samples were then dried to completeness under a gentle stream of nitrogen gas heated to 60 °C, then reconstituted with 100 μL of 50:50 methanol/water before being vortexed and sealed. 10 μL of the extract was injected into the LC–MS/MS. Chromatographic separation was achieved using a CORTECS™ core–shell C18 50 × 2.1 mm, 2.7 µm, reversed-phase (Waters Corp., Milford, MA, USA) column heated at 30˚C. Mobile phases used were (A) water and (B) methanol in 0.1% formic acid, pumped at the flow rate of 0.4 mL/min in 70:30% (A:B), gradually increased to 100% (B) then returned to the starting gradient at 4 min. Tandem mass spectrometry detection was based on the mass-to-charge (m/z) precursor to product ion transitions specific to each compound: cortisol (363.3 > 121.2) and cortisol-d4 (367.3 > 121.2) as an internal standard. The inter-assay CV for serum cortisol was < 6.0% across the assay range of 0.3–806 nmol/L.

Urine normetanephrine was extracted using solid phase extraction method (Chromsystems Biogenic amino #80,600, München, Germany) as per the manufacturer’s instruction. The assay was calibrated using human urine-based calibration standards and controls (Chromsystems) that are traceable to certified reference materials. m/z transitions were: normetanephrine (166.1 > 106.1) and normetanephrine-d3 (169.1 > 109.1) as internal standard. The inter-assay CV for normetanephrine was 3.4–6.2% across the assay range of 20.5–10,311 nmol/L. Urine results obtained from LC–MS/MS analysis were adjusted for variations in renal function by dividing by urine creatinine. Urine creatinine was analysed using Roche 2nd generation kinetic colorimetric assay based on the Jaffé method performed on the COBAS® C501 analyser (Roche, Burgess Hill, UK). The inter-assay CV was < 2.1%. The final results are expressed as nmol/L per mmol/L creatinine.

### Serum C-Reactive Protein high sensitive (hs-CRP)

Serum hs-CRP was measured using a particle-enhanced immunoturbidimetric assay analysed on the COBAS® C501 analyser (Roche, Burgess Hill, UK). The inter-assay CV was < 2.6% across the assay working range 1.43–190 nmol/L.

### Data analysis

Data analysis was performed with SPSS (IBM SPSS Statistics for Windows, Version 27.0. Armonk, NY: IBM Corp). Normality was assessed by visually inspecting histograms and skewness and kurtosis and homogeneity of variance for linear mixed models by plotting the residuals against the predicted values. Variables collected via PSG, actigraphy, and subjective sleep diaries as well as physical activity indices were measured with paired t-tests; any variables not normally distributed were analysed with non-parametric Wilcoxon signed rank tests. Actigraphy and subjective sleep data is an average of the 7 supplementation nights. Core temperature, KSS, fatigue, and all cognitive function and blood outcomes were analysed with linear mixed models. Mixed models were preferred to repeated measures ANOVAs because they better account for missing data. Time, condition (CP vs. CON), and time*condition interaction effects were included as fixed factors, and participant as a random factor. Models were run using the keep it maximal approach recommended by Barr et al. [69]; therefore, random intercepts and random slopes were initially included for all within-subject variables. If convergence wasn’t achieved, then the random effect’s structure was simplified. The covariance type was scaled identity and the model estimation was restricted maximum likelihood. Post hoc tests were adjusted for multiple tests using the Bonferroni correction. Intra and inter-rater reliability for PSG variables were assessed with intraclass correlation coefficients (ICC), using absolute agreement and a two way mixed-effects model (as in SPSS); values < 0.50 were considered to have poor reliability, 0.50–0.75 moderate reliability, 0.76–0.90 good reliability, and > 0.90 excellent reliability [70]. P < 0.05 was considered statistically significant. Hedges* g* effect sizes are reported for normally distributed data; rank biserial correlations (*r*_*rb*_) are reported for non-normally distributed data. 95% confidence intervals (CI) are presented for the difference in means (CON - CP in all tables) or, if non-parametric tests were performed, the median of the differences, using the Hodges-Lehman estimator.

## Results

Three participants were excluded at screening because they scored < 6 on the AIS; one participant dropped out after contracting COVID-19. In total, 13 volunteers completed the full study.

### Polysomnography

For intrarater ICC values, apart from N3 (%) which showed moderate reliability (0.61–0.64), all other variables showed good reliability (≥ 0.83). For interrater ICC values, all variables had good reliability (≥ 0.86), apart from N2 (%), which showed moderate reliability (0.58).

Table 2 displays the data collected via PSG. We failed to get traces for 1 participant and therefore this data is for 12 participants. The data presented is from night 7 of the 7-day supplementation period. During the CON and CP trials participants went to bed at 22:57:13 ± 01:07:14 and 22:50:19 ± 01:09:11, respectively; they woke at 07:10:36 ± 00:47:20 and 07:02:54 ± 00:53:05, respectively. The analysis revealed that participants had less awakenings during the CP trial (P < 0.05).

### Subjective sleep diary data

Subjective SOL did not differ between CP (19.9 ± 6.6 min) and CON (19.5 ± 14.6 min) (P = 0.972; *r*_*rb*_ = 0.010; 95% CI -4.64 to 6.14). Frequency of awakenings was significantly lower in CP (1.3 ± 1.5 counts) vs. CON (1.9 ± 0.6 counts) (P = 0.023; *r*_*rb*_ = 0.744; 95% CI -0.85 to -0.07) but wake length did not differ (CON = 10.3 ± 7.7 vs. CP = 9.6 ± 7.6) (P = 0.694; *r*_*rb*_ = 0.102; 95% CI -2.14 to 3.86).

### Actigraphy sleep data

Sleep variables derived from actigraphs are presented in Table 3. There were no significant differences for any variable (P > 0.05).

**Table 3:** Sleep variables for the control and collagen peptides trials, as measured by wristwatch actigraphy

Variable	Control	Collagen peptides	P value	Effect size	95% CI
Time in bed (min)	488.1 ± 35.3	491.1 ± 43.9	0.710	0.102	-20.1 to 14.1
Total sleep time (min)	382.4 ± 35.8	384.8 ± 37.1	0.654	0.123	-14.1 to 9.20
Sleep latency (min)	10.9 ± 7.7	8.8 ± 5.8	0.155	0.407	-0.90 to 5.06
Sleep efficiency (%)	78.7 ± 7.7	78.9 ± 8.4	0.894	0.036	-2.62 to 2.31
Wake after sleep onset (min)	94.8 ± 37.3	97.5 ± 44.1	0.735	0.093	-17.9 to 13.0
Average wake length (min)	3.34 ± 1.00	3.14 ± 0.83	0.423	0.223	-0.35 to 0.78

Data used for analysis is average of 7 nights. 95% CI, 95 confidence interval for the difference in means. Effect size is Hedges *g*

### Subjective sleep quality and fatigue

Table 4 shows the results from subjective surveys of sleepiness (KSS), fatigue, and sleep. There was a time effect for the KSS (P < 0.001) but there were no significant differences between supplements at any time-point (condition effect: P = 0.303; condition*time interaction effect; P = 0.217). Fatigue was higher mid-day than in the morning (P < 0.001) but there were no between trial differences (condition effect: P = 0.709; condition*time interaction effect; P = 0.275). There were no differences in subjective sleep quality between the CON and CP trials (P = 0.862; *g* = 0.048; 95 CI -0.28 to 0.24).

**Table 4:** Subjective sleep quality, sleepiness, and fatigue scales for the control and collagen peptides trials

Variable	Control	Collagen peptides
KSS-AM (AU)	5.0 ± 0.8	4.9 ± 0.8
KSS-MID (AU)	3.5 ± 0.9**a**	3.5 ± 0.7**a**
KSS-PM (AU)	5.9 ± 0.8**b**	5.3 ± 1.0**b**
Fatigue-AM (mm)	48.2 ± 12.8	49.3 ± 12.6
Fatigue-MID (mm)	63.3 ± 14.9**b**	62.3 ± 13.0**b**
Fatigue-PM (mm)	39.5 ± 8.8	43.3 ± 11.3
Sleep quality (AU)	4.4 ± 0.4	4.5 ± 0.5

AU = arbitrary units; KSS = 7-day average for Karolinska Sleepiness Scale; AM = recorded 30 min after waking; MID = recorded at 14:00; PM = recorded 1 h pre-bed

a = significantly lower than AM (P < 0.05)

b = significantly higher than AM

### Cognitive function

Table 5 shows the results from the cognitive function tests. There were time effects for average and fast, choice reaction time, which decreased Post (P < 0.05) but no condition or interaction effects (P > 0.05). There was a time*condition interaction effect for correct responses on the baseline Stroop test (P = 0.007) with *post-hoc* tests revealing that participants responses were more accurate in CP vs. CON (P = 0.009; *g* = 0.573; 95% CI 0.01 to 0.04).

**Table 5:** Cognitive function tests the evening before (PRE) and morning after (POST) the main trial (day 7)

Variable	Control	CP	P value Time; condition; Interaction
Simple reaction time^Av^ (ms)			0.310; 0.743; 0.424
Pre	306 ± 31	313 ± 33	
Post	319 ± 40	317 ± 29	
Simple reaction time^FAST^ (ms)			0.633; 0.435; 0.669
Pre	260 ± 43	263 ± 68	
Post	257 ± 39	247 ± 73	
Choice reaction time^AV^ (ms)			0.030; 0.138; 0.164
Pre	397 ± 24	414 ± 28	
Post	389 ± 33	392 ± 26	
Choice reaction time^FAST^ (ms)			< 0.001; 0.819; 0.770
Pre	333 ± 14	335 ± 20	
Post	320 ± 23	320 ± 26	
Digit span (AU)			0.670; 0.094; 0.206
Pre	8 ± 2	8 ± 1	
Post	8 ± 1	8 ± 1	
Stroop test BL^AV^ (ms)			0.152; 0.510; 0.803
Pre	667 ± 86	682 ± 105	
Post	642 ± 96	652 ± 82	
Stroop test IF^AV^ (ms)			0.232; 0.621; 0.752
Pre	900 ± 171	887 ± 200	
Post	859 ± 179	832 ± 196	
Stroop test BL^CR^ (AU)*			0.720; 0.285; 0.007
Pre	0.99 ± 0.02	0.98 ± 0.03	
Post	0.97 ± 0.05	1.00 ± 0.00	
Stroop test IF^CR^ (AU)			0.649; 1.000; 0.789
Pre	0.94 ± 0.04	0.95 ± 0.07	
Post	0.95 ± 0.04	0.95 ± 0.09	

*AV* average time; *FAST* fastest time, *BL* baseline, *IF* interference, *CR* correct responses, *AU* arbitrary units

*Interaction effect; significantly higher number of correct responses in the CP vs. control condition in the morning

### Physical activity

Daily training load during the CON and CP trials was 419 ± 214 and 388 ± 269 AU, respectively; these were not significantly different (P = 0.578; *g* = 0.154; 95% CI -85.62 to 146.55). There were also no significant differences in actigraphy derived moderate to vigorous activity during the CON (1210 ± 395 min) and CP trials (1228 ± 416 min) (P = 0.653; *g* = 0.124; 95% CI -103.18 to 67.18).

### Core temperature

**Fig. 1:**
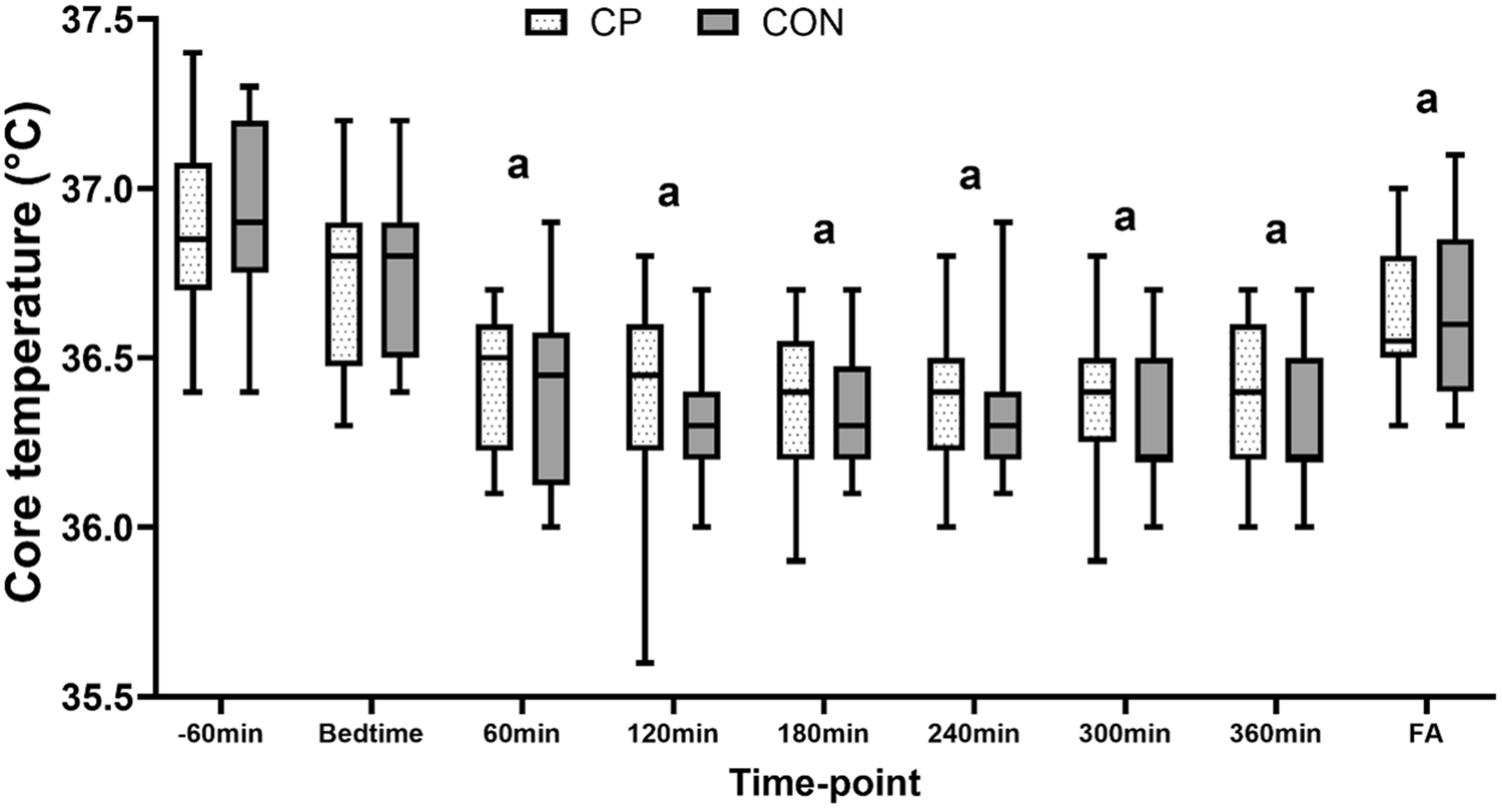
Changes in core temperature during the main trial night (night 7) in the control and collagen peptides (CP) conditions. FA, final awakening/lights on. a = significantly different to -60 min

Core temperature recordings were missing at random for 7/117 time-points in CON and CP. As shown in Fig. 1**,** there was a time effect (P < 0.001) whereby core temperature decreased over-time, but there was no condition (P = 0.409) or condition*time interaction effects (P = 0.728).

### Biological samples

Results from the blood and urine analysis are displayed in Table 6. Due to issues with analysis, 7/52 samples were not analysed for normetanephrine. There was a time effect for cortisol (P < 0.001), which increased Post in both conditions (P < 0.001) but there were no significant condition or interaction effects. There were no time, condition or interaction effects for IL-6, hs-CRP or normetanephrine:creatinine ratio (P > 0.05).

**Table 6:** Interleukin-6 (IL-6), cortisol, high sensitivity CRP (hs-CRP) and normetanephrine:creatinine ratio the evening before (Pre) and morning after (Post) the main trial (night 7) in the control and collagen peptides (CP) conditions

Variable	Control	CP	P value Time; condition; interaction
IL-6 (pg/ml)			
Pre	1.32 ± 2.11	0.67 ± 0.28	0.935; 0.438; 0.127
Post	0.95 ± 0.76	1.09 ± 1.29	
Cortisol (nmol/L)			
Pre	220.8 ± 70.9	245.6 ± 81.0	< 0.001; 0.310; 0.771
Post	353.6 ± 79.5	370.5 ± 96.4	
Hs-CRP (nmol/L)			
Pre	4.11 ± 3.63	3.89 ± 2.04	0.471; 0.666; 0.554
Post	3.61 ± 3.29	3.29 ± 2.04	
Normetanephrine (nmol/L per mmol/L of creatinine)			
Pre	8.63 ± 2.24	8.72 ± 2.95	0.974; 0.528; 0.445
Post	8.92 ± 2.68	8.55 ± 2.65	

*AU* arbitrary units

## Discussion

The main findings of this study are that 7 days of CP supplementation; 1) reduced awakenings, as measured objectively by PSG, and subjectively via sleep diaries, and 2) improved cognitive performance, as measured by the Stroop test. This is the first study to show that CP supplementation may reduce sleep fragmentation in athletic males and suggests CP could be used as a non-pharmacological strategy to enhance sleep quality in athletes, and potentially other populations with sleep complaints.

We found no effect of CP supplementation on our primary outcomes (SOL and SE) or sleep architecture, but did find that CP reduced awakenings, as measured objectively by PSG and subjectively with sleep diaries. Our PSG data showed that another key indicator of sleep fragmentation, WASO, was ~ 5.5 min lower with CP (Table 2), and sleep stage changes were ~ 12% less frequent, but neither were statistically significant changes, and the effect sizes were small. To our knowledge, no other study has examined the impact of CP on markers of sleep quality using PSG for comparisons. Interestingly though, a study in male cyclists used CP as a low tryptophan control to examine the effects of α-lactalbumin on actigraphy derived indices of sleep quality [71]. They found no benefits of α-lactalbumin compared to CP on various measures of sleep quality. One previous study [19] measured the effects of a single dose of glycine (3 g) on polysomnographic sleep and while WASO and SOL were reduced in adults with sleep complaints, awakenings were not recorded. Interestingly, this study [19] and others [51, 72] reported that acute glycine intake enhanced subjective sleep quality and/or reduced subjective sleepiness and fatigue. This contrasts to our study, where we found no effect of CP supplementation on these subjective outcomes. This discrepancy in our findings compared to the aforesaid studies is probably related to the variation in study design; they provided glycine as opposed to CP, their populations were older and non-athletic (24–61 years) and they used different scales and surveys to measure sleepiness and fatigue, at different times of day. Importantly, these studies [19, 51, 72] were also single blinded, which can lead to more favourable results for self-reported outcomes [73]. Irrespective of the precise reasons, given that nocturnal awakenings are associated with poor sleep [74], dysregulated metabolism [75] and increased blood pressure [76], more research is warranted on the effects of CP supplementation on sleep quality and health outcomes.

The mechanisms by which CP reduced awakenings are unclear. The main mechanism by which we hypothesized CP would modulate sleep quality and architecture was related to its high glycine content and effect on circadian rhythms. Indeed, Kawai et al., [18] showed that exogenous glycine improves sleep by activating NMDA receptors in the SCN, which regulates circadian rhythms and lowers nocturnal core temperature [77]. In our study, however, despite decreasing during the night (Fig. 1), core temperature was no different in the control and CP trials. It is possible that our sample size was too small to detect small differences in core temperature between the two conditions, especially given the high inter-subject variability. We also speculated that CP may attenuate inflammation, which is associated with sleep complaints [78–80], but pro-inflammatory cytokines and endocrine markers were no different between the control and CP groups (Table 6). This suggests not only that CP had no direct effect on these markers, but the reduced awakenings also had no effect on these markers. An alternative mechanistic explanation for our findings is that CP increased BDNF levels or other nerve growth factors associated with sleep, as shown in animal brains [25, 26]; however, these were not measured in this study. Nevertheless, we would also expect the aforesaid mechanisms to influence other markers of sleep quality, not just awakenings, as in this study. Thus, it is not clear how or why CP modified awakenings but no other markers of sleep quality, and this should be a focus of future research.

While CP had no effect on tests of RT or short-term memory, it did increase the proportion of correct responses on the baseline Stroop test the morning after the main PSG trial night. Our results are in partial agreement with previous studies examining the effects of glycine on sleep and subsequent cognitive performance. Yamadera and colleagues [19] found no effect of glycine intake on daytime RT but an increase in the proportion of correct responses in a memory recognition task. In Bannai et al., 3 g of glycine before bedtime reduced next day sleepiness; simple RT and memory recognition were unaffected, but RT was faster in the glycine trial during a psychomotor vigilance test [72]. The improvements in some aspects of cognition in these studies and ours could be a positive outcome of the improved sleep with CP, as poor sleep diminishes cognition [81], including Stroop test performance [82]. However, independent of sleep quality, 4 weeks of collagen hydrolysates (5 g/day) was recently shown to enhance cognitive function in older adults [83]. There is also some mechanistic support for CP to affect brain function in regions important for cognition. In mice, prolyl-hydroxyproline peptides, derived from oral collagen hydrolysate ingestion, stimulated neurogenesis in the hippocampus [25, 26, 84], a brain region that contributes to cognitive function [85]. In addition, glycine alone has been shown to improve cognition in some studies [86, 87], ostensibly by activating NMDA receptors, now a promising target in mood disorders [88]. Therefore, we cannot rule out that CP enhanced cognitive performance, independent of the differences in sleep fragmentation.

It is unclear why CP only influenced baseline Stroop test performance; previous studies do not suggest that the Stroop test, which chiefly measures the ability to inhibit cognitive interference, is more sensitive to changes in sleep quality than tests of RT or other aspects of cognition [89–91]. It is possible that the Stroop test is more sensitive to amino acid ingestion than the other cognitive tests employed in this study, but we are unaware of evidence to support this. As brain imaging shows that Stroop interference is linked to activity in the medial and lateral surfaces of the frontal lobes [92, 93], perhaps CP had an impact on these specific brain regions more than those associated with simple RT or memory; however, this needs confirmation with additional research. Irrespective of the precise mechanisms, the improvement in Stroop test performance suggests CP could enhance selective attention and executive function, which could translate to improved focus and an improved ability to process competing information/multitask.

This study has many strengths. Firstly, sleep was assessed objectively using the gold standard PSG method. Furthermore, we familiarised participants to wearing the PSG equipment with a pre-intervention familiarisation week and then mitigated any bias introduced with a first night effect [94] by recording PSG for 2 consecutive nights and discarding the first night from analysis. Secondly, the PSG analysis had excellent intra and interrater reliability for our primary outcomes, and most secondary outcomes, including awakenings (ICC =  ≥ 0.94). We acknowledge that because our participants were young healthy males with self-reported sleep complaints, these findings may not translate to sedentary or older adults, females, or patients with objectively diagnosed sleep disorders. Nevertheless, our results suggest CP has the potential to influence sleep and cognition in these populations as well, and perhaps to an even greater extent in those with insomnia or other sleep related conditions. Given that sleep fragmentation is a key feature of insomnia and sleep apnoea [95], CP could serve as an adjuvant strategy to other lifestyle or psychological interventions currently used to treat sleep disorders. Another possible limitation is that none of our primary outcomes were influenced by CP, only our secondary outcomes. Future studies could test the effect of different CP doses on sleep quality, over shorter or longer periods, and in a range of populations.

In conclusion, our findings demonstrate that 7 days of CP supplementation (15 g/day) reduced nocturnal awakenings and improved Stroop test cognitive performance. While these findings should be interpreted with caution until replicated by future studies, they suggest CP supplements could enhance sleep quality in athletic populations with sleep complaints. As athletes often report lower sleep quality than the general population, especially after late night competitions, strategic intake of CP could mitigate the potentially deleterious effects of poor sleep on psychomotor performance.

## Data Availability

Not available.

## References

[1] Pavlova K, M, Latreille V, (2019). Sleep disorders. Am J Med.

[2] Calem M, Bisla J, Begum A (2012). Increased prevalence of insomnia and changes in hypnotics use in england over 15 years: analysis of the 1993, 2000, and 2007 national psychiatric morbidity surveys. Sleep.

[3] Leger D, Guilleminault C, Dreyfus JP (2000). Prevalence of insomnia in a survey of 12 778 adults in France. J Sleep Res.

[4] Roth T (2007). Insomnia: definition, prevalence, etiology, and consequences. J Clin Sleep Med.

[5] Edinger JD, Arnedt JT, Bertisch SM (2021). Behavioral and psychological treatments for chronic insomnia disorder in adults: an American Academy of Sleep Medicine clinical practice guideline. J Clin Sleep Med.

[6] Riemann D, Baglioni C, Bassetti C (2017). European guideline for the diagnosis and treatment of insomnia. J Sleep Res.

[7] Doherty R, Madigan S, Warrington G, Ellis J (2019). Sleep and nutrition interactions: implications for athletes. Nutrients.

[8] Howatson G, Bell PG, Tallent J (2012). Effect of tart cherry juice (*Prunus cerasus*) on melatonin levels and enhanced sleep quality. Eur J Nutr.

[9] Doherty R, Madigan S, Nevill A (2023). The impact of kiwifruit consumption on the sleep and recovery of elite athletes. Nutrients.

[10] Afaghi A, O’Connor H, Chow CM (2007). High-glycemic-index carbohydrate meals shorten sleep onset. Am J Clin Nutr.

[11] Vlahoyiannis A, Aphamis G, Andreou E (2018). Effects of high vs low glycemic index of post-exercise meals on sleep and exercise performance: a randomized, double-blind, Counterbalanced Polysomnographic Study. Nutrients.

[12] Gratwicke M, Miles K, Clark B, Pumpa K (2022). The effect of α-lactalbumin consumption on sleep quality and quantity in female rugby union athletes: a field-based study. Biol Sport.

[13] Ong JN, Hackett DA, Chow C-M (2017). Sleep quality and duration following evening intake of alpha-lactalbumin: a pilot study. Biol Rhythm Res.

[14] Bravo R, Matito S, Cubero J (2013). Tryptophan-enriched cereal intake improves nocturnal sleep, melatonin, serotonin, and total antioxidant capacity levels and mood in elderly humans. Age.

[15] Markus CR, Jonkman LM, Lammers JH (2005). Evening intake of α-lactalbumin increases plasma tryptophan availability and improves morning alertness and brain measures of attention. Am J Clin Nutr.

[16] Pérez-Cruet J, Chase TN, Murphy DL (1974). Dietary regulation of brain tryptophan metabolism by plasma ratio of free tryptophan and neutral amino acids in humans. Nature.

[17] Claustrat B, Brun J, Chazot G (2005). The basic physiology and pathophysiology of melatonin. Sleep Med Rev.

[18] Kawai N, Sakai N, Okuro M (2015). The sleep-promoting and hypothermic effects of glycine are mediated by nmda receptors in the suprachiasmatic nucleus. Neuropsychopharmacol.

[19] Yamadera W, Inagawa K, Chiba S (2007). Glycine ingestion improves subjective sleep quality in human volunteers, correlating with polysomnographic changes: effects of glycine on polysomnography. Sleep Biol Rhythms.

[20] Ito Y, Takahashi S, Shen M (2014). Effects of L-serine ingestion on human sleep. Springerplus.

[21] Gratwicke M, Miles KH, Pyne DB (2021). Nutritional interventions to improve sleep in team-sport athletes: a narrative review. Nutrients.

[22] Halson SL (2014). Sleep in elite athletes and nutritional interventions to enhance sleep. Sports Med.

[23] Halson SL, Shaw G, Versey N (2020). Optimisation and validation of a nutritional intervention to enhance sleep quality and quantity. Nutrients.

[24] León-López A, Morales-Peñaloza A, Martínez-Juárez VM (2019). Hydrolyzed collagen—sources and applications. Molecules.

[25] Mizushige T, Nogimura D, Nagai A (2019). Ginger-degraded collagen hydrolysate exhibits antidepressant activity in mice. J Nutr Sci Vitaminol.

[26] Nogimura D, Mizushige T, Taga Y (2020). Prolyl-hydroxyproline, a collagen-derived dipeptide, enhances hippocampal cell proliferation, which leads to antidepressant-like effects in mice. FASEB J.

[27] Kushikata T, Fang J, Krueger JM (1999). Brain-derived neurotrophic factor enhances spontaneous sleep in rats and rabbits. Am J Physiol Regulat Integr Compar Physiol.

[28] Flores KR, Viccaro F, Aquilini M (2020). Protective role of brain derived neurotrophic factor (BDNF) in obstructive sleep apnea syndrome (OSAS) patients. PLoS ONE.

[29] Schmitt K, Holsboer-Trachsler E, Eckert A (2016). BDNF in sleep, insomnia, and sleep deprivation. Ann Med.

[30] Suzuki G, Tokuno S, Nibuya M (2014). Decreased plasma brain-derived neurotrophic factor and vascular endothelial growth factor concentrations during military training. PLoS ONE.

[31] Zielinski MR, Kim Y, Karpova SA (2014). Chronic sleep restriction elevates brain interleukin-1 beta and tumor necrosis factor-alpha and attenuates brain-derived neurotrophic factor expression. Neurosci Lett.

[32] Driller MW, Dixon ZT, Clark MI (2017). Accelerometer-based sleep behavior and activity levels in student athletes in comparison to student non-athletes. Sport Sci Health.

[33] Swinbourne R, Gill N, Vaile J, Smart D (2016). Prevalence of poor sleep quality, sleepiness and obstructive sleep apnoea risk factors in athletes. Eur J Sport Sci.

[34] Gupta L, Morgan K, Gilchrist S (2017). Does elite sport degrade sleep quality? A systematic review. Sports Med.

[35] Leduc C, Tee J, Weakley J (2020). The quality, quantity, and intraindividual variability of sleep among students and student-athletes. Sports Health.

[36] Mah CD, Kezirian EJ, Marcello BM, Dement WC (2018). Poor sleep quality and insufficient sleep of a collegiate student-athlete population. Sleep Health.

[37] Lo H, Leung J, Chau KY (2017). Factors affecting sleep quality among adolescent athletes. Sports Nutrition and Therapy.

[38] Oda S, Shirakawa K (2014). Sleep onset is disrupted following pre-sleep exercise that causes large physiological excitement at bedtime. Eur J Appl Physiol.

[39] Aloulou A, Duforez F, Bieuzen F, Nedelec M (2020). The effect of night-time exercise on sleep architecture among well-trained male endurance runners. J Sleep Res.

[40] Praet SFE, Purdam CR, Welvaert M (2019). Oral supplementation of specific collagen peptides combined with calf-strengthening exercises enhances function and reduces pain in achilles tendinopathy patients. Nutrients.

[41] Shaw G, Lee-Barthel A, Ross ML (2017). Vitamin C–enriched gelatin supplementation before intermittent activity augments collagen synthesis. Am J Clin Nutr.

[42] Clifford T, Ventress M, Allerton DM (2019). The effects of collagen peptides on muscle damage, inflammation and bone turnover following exercise: a randomized, controlled trial. Amino Acids.

[43] Zdzieblik D, Jendricke P, Oesser S (2021). The influence of specific bioactive collagen peptides on body composition and muscle strength in middle-aged, untrained men: a randomized controlled trial. Int J Environ Res Public Health.

[44] Faul F, Erdfelder E, Lang AG, Buchner A (2007) G*Power 3: A flexible statistical power analysis program for the social, behavioral, and biomedical sciences. In: Behavior Research Methods 10.3758/bf03193146 17695343

[45] Baskett JJ, Broad JB, Wood PC (2003). Does melatonin improve sleep in older people?.

[46] Taylor G, Leonard A, Tang JCY (2022). The effects of collagen peptides on exercise-induced gastrointestinal stress: a randomized, controlled trial. Eur J Nutr.

[47] Notley SR, Meade RD, Kenny GP (2021). Time following ingestion does not influence the validity of telemetry pill measurements of core temperature during exercise-heat stress: the journal Temperature toolbox. Temperature.

[48] Riera F, Bellenoue S, Fischer S, Méric H (2021). Impact of a cold environment on the performance of professional cyclists: a pilot study. Life (Basel).

[49] Bongers CCWG, Daanen H, a. M, Bogerd CP, (2018). Validity, reliability, and inertia of four different temperature capsule systems. Med Sci Sports Exerc.

[50] Goods PSR, Maloney P, Miller J (2023). Concurrent validity of the CORE wearable sensor with BodyCap temperature pill to assess core body temperature during an elite women’s field hockey heat training camp. Eur J Sport Sci.

[51] Inagawa K, Hiraoka T, Kohda T (2006). Subjective effects of glycine ingestion before bedtime on sleep quality. Sleep Biol Rhythms.

[52] Alcock RD, Shaw GC, Tee N, Burke LM (2019). Plasma amino acid concentrations after the ingestion of dairy and collagen proteins, in healthy active males. Frontiers in Nutrition.

[53] Soldatos CR, Dikeos DG, Paparrigopoulos TJ (2000). Athens Insomnia Scale: validation of an instrument based on ICD-10 criteria. J Psychosom Res.

[54] Chiu H-Y, Chang L-Y, Hsieh Y-J, Tsai P-S (2016). A meta-analysis of diagnostic accuracy of three screening tools for insomnia. J Psychosom Res.

[55] Dickinson RK, Hanrahan SJ (2009). An investigation of subjective sleep and fatigue measures for use with elite athletes. J Clin Sport Psychol.

[56] Horne JA, Ostberg O (1976). A self-assessment questionnaire to determine morningness-eveningness in human circadian rhythms. Int J Chronobiol.

[57] Foster C, Florhaug JA, Franklin J (2001). A new approach to monitoring exercise training. J Strength Cond Res.

[58] Cole RJ, Kripke DF, Gruen W (1992). Automatic sleep/wake identification from wrist activity. Sleep.

[59] Freedson PS, Melanson E, Sirard J (1998). Calibration of the computer science and applications, inc. accelerometer. Med Sci Sports Exerc.

[60] Bruyneel M, Libert W, Ameye L, Ninane V (2015). Comparison between home and hospital set-up for unattended home-based polysomnography: a prospective randomized study. Sleep Med.

[61] Campbell AJ, Neill AM (2011). Home set-up polysomnography in the assessment of suspected obstructive sleep apnea: Home polysomnography and diagnosis of OSA. J Sleep Res.

[62] AASM Scoring Manual - American Academy of Sleep Medicine. In: American Academy of Sleep Medicine – Association for Sleep Clinicians and Researchers. https://aasm.org/clinical-resources/scoring-manual/. Accessed 24 Nov 2022

[63] Åkerstedt T, Gillberg M (1990). Subjective and objective sleepiness in the active individual. Int J Neurosci.

[64] Bannai M, Kawai N (2012). New therapeutic strategy for amino acid medicine: glycine improves the quality of sleep. J Pharmacol Sci.

[65] Ostrosky-Solís F, Lozano A (2006). Digit span: effect of education and culture. Int J Psychol.

[66] Scarpina F, Tagini S (2017) The Stroop Color and Word Test. Frontiers in Psychology 8:10.3389/fpsyg.2017.00557 PMC5388755 28446889

[67] Wright BC (2017). What stroop tasks can tell us about selective attention from childhood to adulthood. Br J Psychol.

[68] Faraut B, Boudjeltia KZ, Vanhamme L, Kerkhofs M (2012). Immune, inflammatory and cardiovascular consequences of sleep restriction and recovery. Sleep Med Rev.

[69] Barr DJ, Levy R, Scheepers C, Tily HJ (2013). Random effects structure for confirmatory hypothesis testing: keep it maximal. J Mem Lang.

[70] Koo TK, Li MY (2016). A guideline of selecting and reporting intraclass correlation coefficients for reliability research. J Chiropr Med.

[71] MacInnis MJ, Dziedzic CE, Wood E (2020). Presleep α-lactalbumin consumption does not improve sleep quality or time-trial performance in cyclists. Int J Sport Nutr Exerc Metab.

[72] Bannai M, Kawai N, Ono K (2012). The effects of glycine on subjective daytime performance in partially sleep-restricted healthy volunteers. Front Neur.

[73] Hróbjartsson A, Emanuelsson F, Skou Thomsen AS (2014). Bias due to lack of patient blinding in clinical trials. A systematic review of trials randomizing patients to blind and nonblind sub-studies. Int J Epidemiol.

[74] Åkerstedt T, Schwarz J, Gruber G (2019). Short sleep—poor sleep? A polysomnographic study in a large population-based sample of women. J Sleep Res.

[75] Stamatakis KA, Punjabi NM (2010). Effects of sleep fragmentation on glucose metabolism in normal subjects. Chest.

[76] Chouchou F, Pichot V, Pépin JL (2013). Sympathetic overactivity due to sleep fragmentation is associated with elevated diurnal systolic blood pressure in healthy elderly subjects: the PROOF-SYNAPSE study. Eur Heart J.

[77] Buijs RM, Guzmán Ruiz MA, Méndez Hernández R, Rodríguez Cortés B (2019). The suprachiasmatic nucleus; a responsive clock regulating homeostasis by daily changing the setpoints of physiological parameters. Auton Neurosci.

[78] Dumaine JE, Ashley NT (2015). Acute sleep fragmentation induces tissue-specific changes in cytokine gene expression and increases serum corticosterone concentration. Am J Physiol Regul Integr Comp Physiol.

[79] Dzierzewski JM, Donovan EK, Kay DB (2020). Sleep inconsistency and markers of inflammation. Front Neurol.

[80] Irwin MR, Olmstead R, Carroll JE (2016). Sleep disturbance, sleep duration, and inflammation: a systematic review and meta-analysis of cohort studies and experimental sleep deprivation. Biol Psychiat.

[81] Ferrie JE, Shipley MJ, Akbaraly TN (2011). Change in sleep duration and cognitive function: findings from the whitehall II study. Sleep.

[82] Gevers W, Deliens G, Hoffmann S (2015). Sleep deprivation selectively disrupts top–down adaptation to cognitive conflict in the Stroop test. J Sleep Res.

[83] Koizumi S, Inoue N, Sugihara F, Igase M (2019). Effects of collagen hydrolysates on human brain structure and cognitive function: a pilot clinical study. Nutrients.

[84] Kakoi C, Udo H, Matsukawa T, Ohnuki K (2012). Collagen peptides enhance hippocampal neurogenesis and reduce anxietyrelated behavior in mice. Biomed Res.

[85] Lisman J, Buzsáki G, Eichenbaum H (2017). Viewpoints: how the hippocampus contributes to memory, navigation and cognition. Nat Neurosci.

[86] File SE, Fluck E, Fernandes C (1999). Beneficial effects of glycine (bioglycin) on memory and attention in young and middle-aged adults. J Clin Psychopharmacol.

[87] Schwartz BL, Hashtroudi S, Herting RL (1991). Glycine prodrug facilitates memory retrieval in humans. Neurology.

[88] Peyrovian B, Rosenblat JD, Pan Z (2019). The glycine site of NMDA receptors: a target for cognitive enhancement in psychiatric disorders. Prog Neuropsychopharmacol Biol Psychiatry.

[89] Lim J, Dinges DF (2010). A meta-analysis of the impact of short-term sleep deprivation on cognitive variables. Psychol Bull.

[90] Nebes RD, Buysse DJ, Halligan EM (2009). Self-reported sleep quality predicts poor cognitive performance in healthy older adults. J Gerontol.

[91] Patrick Y, Lee A, Raha O (2017). Effects of sleep deprivation on cognitive and physical performance in university students. Sleep Biol Rhythms.

[92] Langenecker SA, Nielson KA, Rao SM (2004). fMRI of healthy older adults during stroop interference. Neuroimage.

[93] Zoccatelli G, Beltramello A, Alessandrini F (2010). Word and position interference in stroop tasks: a behavioral and fMRI study. Exp Brain Res.

[94] Ding L, Chen B, Dai Y, Li Y (2022). A meta-analysis of the first-night effect in healthy individuals for the full age spectrum. Sleep Med.

[95] Kimoff RJ (1996). Sleep Fragmentation in Obstructive Sleep Apnea. Sleep.

